# Systemic immune inflammation index and all‐cause mortality in chronic kidney disease: A prospective cohort study

**DOI:** 10.1002/iid3.1358

**Published:** 2024-09-10

**Authors:** Meng Jia, Wenli Yuan, Yinqing Chen, Yi Wang, Li Shang, Shisheng Han

**Affiliations:** ^1^ Department of Nephrology, Yueyang Hospital of Integrated Traditional Chinese and Western Medicine Shanghai University of Traditional Chinese Medicine Shanghai China; ^2^ Institute of Science, Technology and Humanities Shanghai University of Traditional Chinese Medicine Shanghai China

**Keywords:** all‐cause mortality, chronic kidney disease, cohort study, NHANES, systemic immune inflammation index

## Abstract

**Background:**

The aim of this study was to investigate the association between systemic immune‐inflammation index (SII) and all‐cause mortality in individuals with chronic kidney disease (CKD).

**Patients and Methods:**

This prospective cohort study was carried out among 9303 participants with CKD from the National Health and Nutrition Examination Survey cycles spanning 1999 to 2018. The mortality data were ascertained by linking participant records to the National Death Index up to December 31, 2019. Complex sampling‐weighted multivariate Cox proportional hazards models were employed to estimate the association between SII level and all‐cause mortality, providing hazard ratios (HR) and 95% confidence intervals (CI). A restricted cubic spline analysis was conducted to explore potential nonlinear correlation. Subgroup analyses and sensitivity analyses were also conducted.

**Results:**

During a median follow‐up period of 86 months, 3400 (36.54%) all‐cause deaths were documented. A distinctive “J”‐shaped relationship between SII level and all‐cause mortality was discerned among individuals with CKD, with the nadir observed at an SII level of 478.93 within the second quartile. After adjusting for potential covariates, the risk of all‐cause mortality escalated by 13% per increment of one standard deviation of SII, once SII exceeded 478.93 (HR = 1.13; 95% CI = 1.08–1.18). An elevated SII was associated with an increased risk of all‐cause mortality among patients with CKD (Q4 vs. Q2: HR = 1.23; 95% CI = 1.01–1.48). Subgroup analyses indicated that the correlation between SII and CKD mortality was particularly pronounced among participants over 60 years old and individuals with diabetes. Sensitivity analyses revealed a linear positive association between SII and all‐cause mortality after removing the extreme 5% outliers of SII.

**Conclusions:**

A distinctive “J”‐shaped relationship between SII level and all‐cause mortality was identified among individuals with CKD. Further research is warranted to validate and expand upon these findings.

## Graphical abstract

This study aimed to investigate the association between systemic immune‐inflammation index (SII) and all‐cause mortality in individuals with chronic kidney disease (CKD). A distinctive “J”‐shaped relationship between SII level and all‐cause mortality was identified among individuals with CKD.

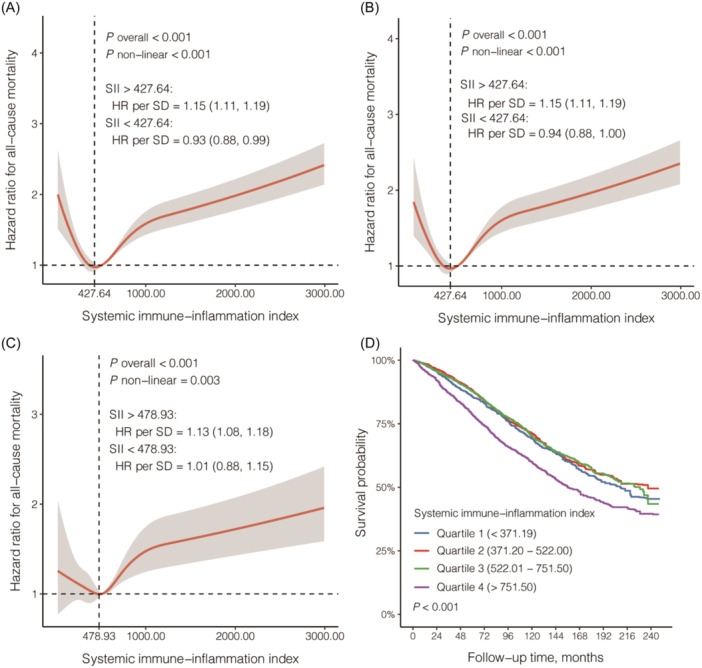

## 1 INTRODUCTION

Chronic kidney disease (CKD) stands as a progressive condition characterized by the gradual decline of renal function, imparting a significantly heightened risk of all‐cause mortality worldwide.^1^ One of the features of CKD is chronic systemic inflammation, driven by various factors, such as oxidative stress, endothelial dysfunction, and immune dysregulation.^2^ Inflammation has been recognized as a pivotal factor in the pathogenesis and progression of CKD.^3^ Furthermore, it is imperative to underscore that sustained low‐grade inflammation is notably linked to an elevated jeopardy of all‐cause mortality within these patients.^4^ In recent years, systemic immune‐inflammation index (SII) has emerged as a potential prognostic marker for various diseases, including cancers, cardiovascular diseases, and kidney diseases.^5^, ^6^ SII is a composite index calculated through the multiplication of platelet count by neutrophil count, and subsequently divided by lymphocyte count.^7^ SII was considered as a reliable measure for the assessment of inflammatory status, which reflected an aggravated systemic inflammatory response, indicating a higher burden of inflammation in the context of CKD.^8^ Previous studies have determined the positive association between SII and CKD incidence.^9^ Moreover, SII was found to be positively associated with increased albuminuria in American adults.^10^ Elevated SII level was also emerged as a harbinger of augmented risk pertaining to diabetic kidney disease (DKD) in individuals with type 2 diabetes.^11^ Noteworthy, SII has been found to be correlated with adverse outcomes among patients with both cardiac disease and CKD. Wang et al. found that high levels of SII led to a 70.3% increase in all‐cause mortality among patients with advanced chronic heart failure accompanied by renal dysfunction.^12^ Recently, Lai et al. demonstrated that increased SII level was associated with a higher risk of all‐cause mortality among patients with CKD undergoing coronary angiography during a median follow‐up of 4.5 years.^13^ However, the association between SII and all‐cause mortality in nationally representative community CKD populations remains limited, and the impact of SII on mortality across different stages of CKD is still unclear. In addition, whether potential factors could influence the association of interest is also unclear. In light of these uncertainties, this study was designed to explore the potential association between SII and all‐cause mortality in patients with CKD through prospective cohort study based on a nationally representative sample of adults in the United States.

## 2 METHODS

### 2.1 Patients and data sources

This is a cohort study reported according to the guideline of strengthening the reporting of observational studies in epidemiology. The data were sourced from the National Health and Nutrition Examination Surveys (NHANES) cycles spanning 1999 to 2018, a comprehensive cross‐sectional survey administered by the National Center for Health Statistics. NHANES was designed to gather information on the representative nutritional and health status of American civilians, utilizing structured interviews, questionnaires, physical examinations, and laboratory tests. NHANES was approved by the National Center for Health Statistics Ethics Review Board, and all participants have signed the informed consent forms.

**Figure 1:**
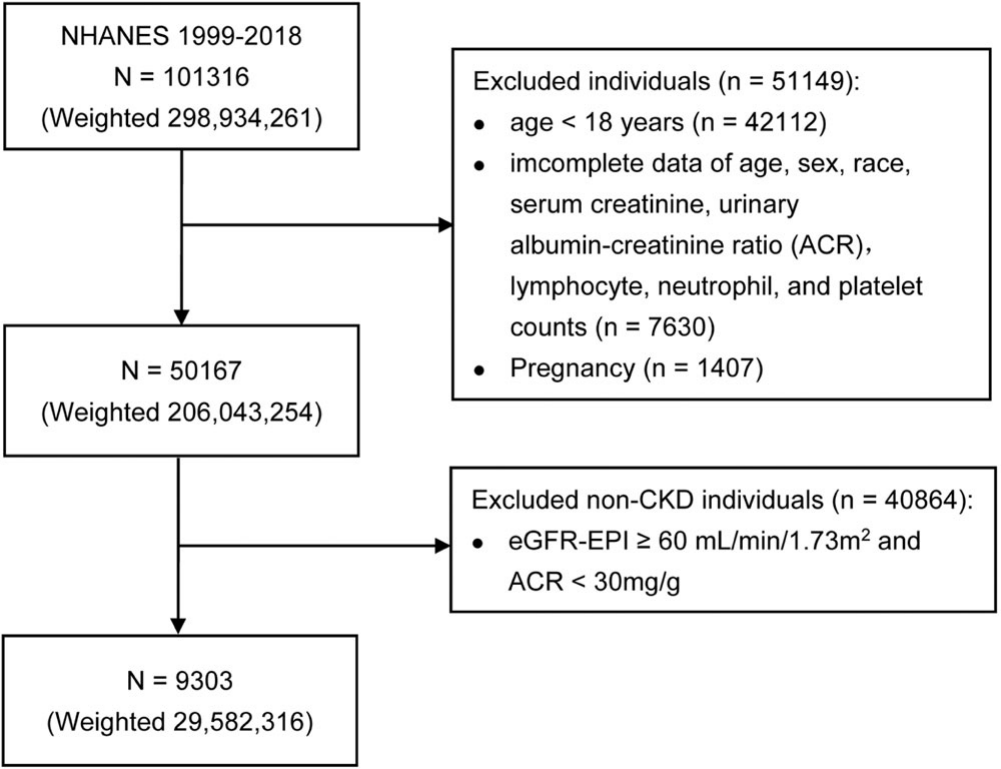
Flowchart of participants selection. ACR, urinary albumin‐to‐creatinine ratio; CKD, chronic kidney disease; eGFR‐EPI, estimated glomerular filtration rate calculated by the CKD‐EPI creatinine equation; NHANES, National Health and Nutrition Examination Surveys.

CKD was defined as an estimated glomerular filtration rate (eGFR) of less than 60 mL/min/1.73 m^2^ calculated by the CKD‐EPI creatinine equation, or a urinary albumin‐to‐creatinine ratio (ACR) equal to or exceeding 30 mg/g.^14^ To precisely identify individuals with CKD and explore the association between SII and CKD in adults, we excluded the following participants: (1) age < 18 years (*n* = 42112); (2) participants with incomplete data on age, sex, race, serum creatinine, ACR, lymphocyte, neutrophil, and platelet counts (*n* = 7630); (3) participants who were currently pregnant (*n* = 1407); and (4) non‐CKD individuals (*n* = 40864). Subsequently, 9303 patients afflicted with CKD were ultimately included for analysis among the 101316 participants from NHANES 1999–2018. The detailed selection process is shown in Figure 1.

### 2.2 Exposure

The complete blood analysis with multi‐part differential counts was performed on the Coulter® DxH 800 analyzer, which can provide the exposure variable required for this study, that is SII, calculated by neutrophil count (unit, 10^3^ cells/uL), lymphocyte count (unit, 10^3^ cells/uL), and platelet count (unit, 10^3^ cells/uL).

### 2.3 Outcome

The mortality status of participants from NHANES 1999–2018 was determined by matching them to the records of the National Death Index (NDI). The public‐use mortality files provide mortality follow‐up data from the date of survey participation through December 31, 2019. We investigated all‐cause mortality among included participants through linking NHANES samples to NDI by each participant's respondent sequence number.

### 2.4 Covariates

Several confounding factors have been previously found to be potentially associated with SII and all‐cause mortality in CKD, and were identified as covariates, such as demographic factors,^15^, ^16^ body mass index (BMI),^17^, ^18^ smoking status,^19^ hypertension,^20^, ^21^ diabetes,^11^, ^22^ dyslipidemia,^23^, ^24^ hyperuricemia,^25^, ^26^ albuminuria,^27^ eGFR,^28^ and mineral metabolism abnormalities.^29^ Demographic factors, such as age, sex, and race, were labeled in household and family‐level information through standardized questionnaires. BMI was calculated as weight in kilograms divided by the square of height in meters. The classification of smoking status was divided into three distinct categories according to self‐reported participant accounts: never smokers, current smokers, and former smokers. Specifically, individuals who had consumed fewer than 100 cigarettes in their lifetime were categorized as never smokers, whereas those who had smoked more than 100 cigarettes and were currently smoking were designated as current smokers. Participants who had smoked more than 100 cigarettes but had quit smoking were classified as former smokers. Hypertension was defined as a self‐reported medical diagnosis of hypertension, or systolic blood pressure equal to or exceeding 140 mmHg, or diastolic blood pressure equal to or exceeding 90 mmHg during the physical examination. Diabetes was defined as a self‐reported medical diagnosis of diabetes, or the attainment of a glycated hemoglobin A1c level equal to or exceeding 7.0%. Serum uric acid (SUA), total cholesterol (TC), triglycerides (TG), low‐density lipoproteins (LDL), total calcium, phosphorus, and parathyroid hormone (PTH), were obtained from the standard biochemistry profile.

### 2.5 Statistical analysis

All the analyses incorporated sample weights, strata, and primary sampling units to assess representative national estimates. Descriptive statistics were presented as numbers (percentages) for categorical variables, and means (standard errors, SE) or medians (interquartile ranges, IQR) for continuous variables as appropriate. The baseline characteristics between the groups stratified by SII quartiles were compared using one‐way ANOVA tests, Kruskal‐Wallis tests, and χ^2^ tests. Complex sampling‐weighted multivariate Cox proportional hazards models were employed to assess the hazard ratios (HR) and their corresponding 95% confidence intervals (CI) of all‐cause mortality among patients with CKD at different SII levels. Three distinct models of Cox proportional hazard regression were conducted in this study. The crude model did not adjust for any covariate. Model 1 was adjusted for age (continues), sex (male or female), and race/ethnicity (Non‐Hispanic White, Non‐Hispanic Black, Mexican American, other Hispanic, or others). Model 2 was further adjusted for additional covariates, including BMI (<25, 25–29.9, or 30 kg/m^2^), smoking status (former, current, or never smoker), hypertension (yes or no), diabetes (yes or no), ACR (≥30 or <30 mg/g), eGFR (continuous), SUA (≥7 or <7 mg/dL), TC (≥240 or <240 mg/dL), TG (≥200 or <200 mg/dL), LDL (≥100 or <100 mg/dL),^30^ total calcium (continuous), phosphorus (continuous), and total leukocyte count (continuous). Missing covariate data was addressed through complete‐case analysis, and samples with missing values were excluded in Model 2. Assumptions of the Cox proportional hazard model were checked using both the Schoenfeld residuals statistical test and graphical method. Model adequacy was further evaluated through the Schoenfeld residuals global test. Additionally, Kaplan‐Meier curves were employed to illustrate differential survival rates for all‐cause mortality based on SII quartiles.

A restricted cubic spline was conducted to investigate the nonlinear correlation between SII and all‐cause mortality of CKD, using five knots placed at the 5%, 27.5%, 50%, 72.5%, and 95% of the SII distribution. Subgroup analyses were executed to discern the association between SII and all‐cause mortality in varying subgroups within the CKD cohort. The stratification factors considered were gender (male or female), age (≥60 or <60 years), race/ethnicity (White or Black or others), eGFR (≥60 or <60 mL/min/1.73 m^2^), ACR (≥30 or <30 mg/g), SUA (≥7 or <7 mg/dL), diabetes (yes or no), hypertension (yes or no), BMI (≥30 or <30 kg/m^2^), and smoking status(never or current/former). In sensitivity analysis, the extreme 5% of SII outliers were excluded to reduce their potential influence. As a part of additional sensitivity analysis, we excluded participants who died within the first years of follow‐up, to minimize the potential reverse causation bias.

All the analyses were conducted using R studio (version 2022.07.2 Build 576, open‐source edition). Two‐sided *p* value less than .05 was considered as statistically significant.

## 3 RESULTS

### 3.1 Baseline characteristics

The baseline characteristics of the included 9303 participants stratified by quartiles of SII in NHANES 1999–2018 are detailed in Table 1. The mean age of the participants was 60.13 years old, of which 52.36% were women. The median and mean values of SII were 522.00 (IQR = 371.20–751.50) and 623.89 (SE = 6.70), respectively. Females comprised 49.30%, 54.36%, 53.65%, and 52.58% of participants in the first, second, third, and fourth quartiles, respectively. Individuals with increased levels of SII also exhibited a higher likelihood of being Non‐Hispanic White (Q4 vs. Q1, 57.84% vs. 34.72%), but conversely, a diminished likelihood of being Non‐Hispanic Black (Q4 vs. Q1, 15.22% vs. 38.05%). Participants characterized by elevated SII values were found to possess heightened ACR [Q4 vs. Q1, 44.12 (16.88, 109.17) vs. 39.29 (10.99, 89.82), mg/g], elevated platelet counts (Q4 vs. Q1, 296.49 ± 2.48 vs. 203.63 ± 1.31, ×10^3^ cells/uL), increased neutrophil counts (Q4 vs. Q1, 6.15 ± 0.06 vs. 3.26 ± 0.03, ×10^3^ cells/uL), and diminished lymphocyte counts (Q4 vs. Q1, 1.67 ± 0.02 vs. 2.75 ± 0.11, ×10^3^ cells/uL). Additionally, patients with elevated SII levels exhibited a higher prevalence of smoking behavior (smoker, Q4 vs. Q1, 54.78% vs. 47.43%). There were no significant differences in serum levels of TC, TG, LDL, SUA, PTH, total calcium, phosphorus, and BMI between different quartiles of SII (*p* > .05), nor in the prevalence of hypertension and diabetes (*p* > .05).

**Table 1:** Baseline characteristics of included participants stratified by quartiles of SII in NHANES 1999–2018.

	Systemic immune‐inflammation index					
Characteristics	Overall	Quartile 1 (4.05–371.19)	Quartile 2 (371.20–522.00)	Quartile 3 (522.01–751.50)	Quartile 4 (>751.50)	*p* value
SII	623.89 ± 6.70	273.62 ± 1.93	443.39 ± 1.21	626.10 ± 2.02	1,152.52 ± 15.60	<.001
Participant (no. death)	9303 (3400)	2578 (793)	2235 (756)	2263 (808)	2227 (1043)	<.001
Age, years	60.13 ± 0.29	60.13 ± 0.51	59.56 ± 0.50	60.28 ± 0.49	60.54 ± 0.58	.329
Sex, female no. (%)	4871 (52.36%)	1271 (49.30%)	1215 (54.36%)	1214 (53.65%)	1171 (52.58%)	.020
Race/ethnicity, no. (%)						<.001
Non‐Hispanic White	4312 (46.35%)	895 (34.72%)	1019 (45.59%)	1110 (49.05%)	1288 (57.84%)	
Non‐Hispanic Black	2336 (25.11%)	981 (38.05%)	543 (24.30%)	473 (20.90%)	339 (15.22%)	
Mexican American	1384 (14.88%)	315 (12.22%)	362 (16.20%)	363 (16.04%)	344 (15.45%)	
Other Hispanic	623 (6.70%)	176 (6.83%)	153 (6.85%)	167 (7.38%)	127 (5.70%)	
Others	648 (6.96%)	211.00 (8.18%)	158 (7.06%)	150 (6.63%)	129 (5.79%)	
CKD stages						0.002
CKD 1	2490 (26.77%)	663 (25.72%)	604 (27.02%)	640 (28.28%)	583 (26.18%)	
CKD 2	2110 (22.68%)	565 (21.92%)	511 (22.86%)	494 (21.83%)	540 (24.25%)	
CKD 3	4236 (45.53%)	1253 (48.60%)	1018 (45.55%)	1,016 (44.90%)	949 (42.61%)	
CKD 4	355 (3.82%)	65 (2.52%)	87 (3.89%)	87 (3.84%)	116 (5.21%)	
CKD 5	112 (1.20%)	32 (1.24%)	15 (0.68%)	26 (1.15%)	39 (1.75%)	
eGFR, mL/min/1.73 m^2^	72.66 ± 0.45	72.48 ± 0.80	73.03 ± 0.79	73.09 ± 0.85	72.02 ± 0.85	.762
ACR, mg/g	41.00 (12.80, 92.30)	39.29 (10.99, 89.82)	37.49 (11.25, 80.48)	43.22 (13.51, 91.25)	44.12 (16.88, 109.17)	<.001
Platelet count, 10^3^ cells/uL	247.28 ± 1.16	203.63 ± 1.31	235.12 ± 1.65	253.87 ± 1.73	296.49 ± 2.48	<.001
Neutrophil count, 10^3^ cells/uL	4.56 ± 0.03	3.26 ± 0.03	4.04 ± 0.04	4.80 ± 0.04	6.15 ± 0.06	<.001
Lymphocyte count, 10^3^ cells/uL	2.12 ± 0.03	2.75 ± 0.11	2.13 ± 0.02	1.94 ± 0.02	1.67 ± 0.02	<.001
SUA, mg/dL	5.96 ± 0.02	5.96 ± 0.04	5.89 ± 0.05	5.99 ± 0.05	5.99 ± 0.05	.228
TC, mg/dL	195.58 ± 0.72	195.99 ± 1.22	195.90 ± 1.25	195.23 ± 1.39	195.19 ± 1.40	.989
TG, mg/dL	169.88 ± 2.25	172.52 ± 4.54	168.82 ± 3.33	172.19 ± 5.57	165.99 ± 3.10	.532
LDL, mg/dL	110.96 ± 0.71	110.84 ± 1.32	113.51 ± 1.69	110.45 ± 1.64	109.06 ± 1.47	.500
Total calcium, mg/dL	9.45 ± 0.01	9.46 ± 0.01	9.47 ± 0.01	9.45 ± 0.01	9.43 ± 0.01	.304
Phosphorus, mg/dL	3.73 ± 0.01	3.73 ± 0.02	3.75 ± 0.01	3.72 ± 0.02	3.73 ± 0.02	.659
PTH, pg/mL	46.00 (34.00, 65.00)	47.00 (34.00, 62.00)	45.00 (32.00, 60.00)	46.00 (36.00, 66.00)	48.00 (34.00, 68.00)	.110
BMI, kg/m^2^	29.81 ± 0.12	29.35 ± 0.19	29.57 ± 0.18	30.33 ± 0.26	29.99 ± 0.21	.099
Hypertension, no. (%)	6379 (68.57%)	1763 (68.39%)	1524 (68.19%)	1553 (68.63%)	1539.00 (69.11%)	.340
Diabetes, no. (%)	2862 (30.76%)	775 (30.06%)	677 (30.29%)	714 (31.55%)	696 (31.25%)	.121
Smoking status						<.001
Current smoker	1537 (16.93%)	410 (16.34%)	345 (15.79%)	400 (18.12%)	382 (17.56%)	
Former smoker	3019 (33.26%)	780 (31.09%)	724 (33.14%)	705 (31.93%)	810 (37.22%)	
Never smoker	4522 (49.81%)	1319 (52.57%)	1116 (51.07%)	1103 (49.95%)	984 (45.22%)	

*Note*: Data were presented as numbers (percentages) for categorical variables, and means (standard errors) or medians (interquartile ranges) for continuous variables as appropriate. Comparisons were conducted using one‐way ANOVA test, Kruskal‐Wallis test, and χ^2^ test.

Abbreviations: ACR, urinary albumin‐to‐creatinine ratio; BMI, body mass index; CKD, chronic kidney disease; eGFR, estimated glomerular filtration rate; LDL, low‐density lipoproteins; PTH, parathyroid hormone; SII, systemic immune‐inflammation index; SUA, serum uric acid; TC, total cholesterol; TG, triglycerides.

### 3.2 Associations of SII and all‐cause mortality in CKD

**Figure 2:**
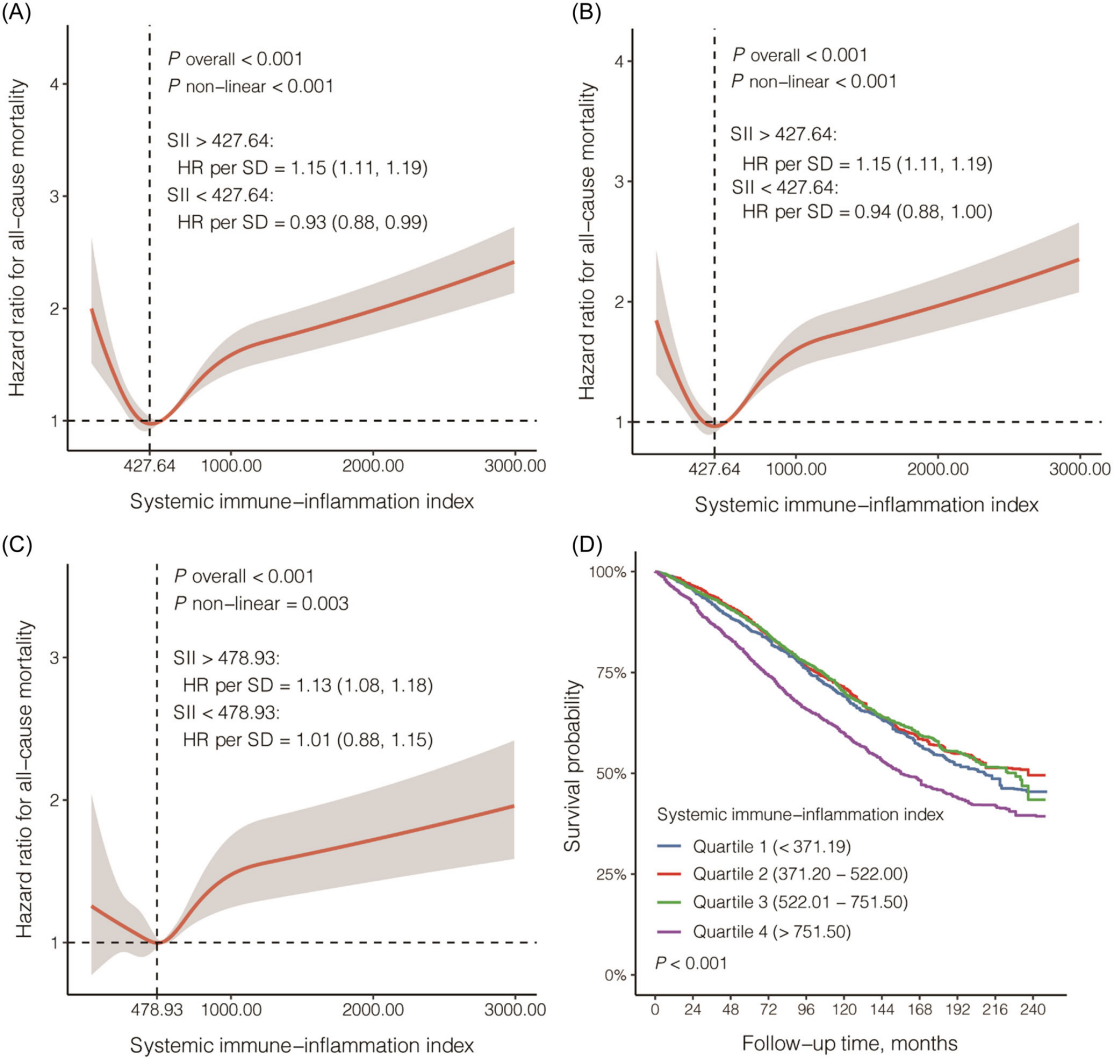
Association between systemic immune‐inflammation index and all‐cause mortality in chronic kidney disease using restricted cubic splines model. (A) Unadjusted model; (B) Model 1, adjusted for age (continues), sex (male or female), and race/ethnicity (non‐Hispanic White, non‐Hispanic Black, Mexican American, other Hispanic, or others); (C) Model 2, further adjusted for additional covariates, including BMI (<25, 25–29.9, or 30 kg/m^2^), smoking status (former, current, or never smoker), hypertension (yes or no), diabetes (yes or no), ACR (≥30 or <30 mg/g) or eGFR (continuous), uric acid (≥7 or <7 mg/dL), cholesterol (≥240 or < 240 mg/dL), triglycerides (≥200 or <200 mg/dL), LDL (≥100 or <100 mg/dL), total calcium (continuous), phosphorus (continuous), and total leukocyte count (continuous); (D) Kaplan Meier curves based on quartiles of systemic immune‐inflammation index. HR, hazard ratio; SD, standard deviation.

During a median of 86 months of follow‐up period, 3400 deaths were documented, yielding an overall survival rate of 63.45%. After checking for assumption and model fitness of Cox proportional hazard model, we found a intricate “J” shaped association between SII and all‐cause mortality in participants with CKD using a restricted cubic splines model, and both the nonlinear tests and overall associations showed statistical differences (Figure 2A–C). As elucidated in Figure 2, the risk of all‐cause mortality initially declined, and subsequently ascending after attaining the nadir of HR. The nadir of risk for all‐cause mortality was realized when SII stood at 427.64 (unadjusted model and model 1) and 478.93 (model 2). In the unadjusted model and model 1, the all‐cause mortality risk amplified by 15% for every increment of one standard deviation (SD = 506.07), once SII exceeded 427.64 (HR per SD = 1.15; 95% CI = 1.11–1.19). A parallel association was echoed in model 2, wherein an SII surpassing 478.93 corresponded to a 13% elevation in the risk of all‐cause mortality per increase of SD (HR per SD = 1.13; 95% CI = 1.08–1.18).

Considering that patients with CKD had the lowest risk of death when SII resided within the second quartile, quartile 2 was designated as the reference to facilitate a comparative assessment of all‐cause mortality risk across diverse quartiles. We found that patients with CKD in higher SII quartile were associated with increased risk of all‐cause death (Q4 vs. Q2 in unadjusted model: HR = 1.50; 95% CI = 1.34–1.68; Q4 vs. Q2 in model 1: HR = 1.47; 95% CI = 1.32–1.63), even after adjusting for all plausible covariates (Q4 vs. Q2 in model 2: HR = 1.23; 95% CI = 1.01–1.48) (Table 2 and Figure 2D).

**Table 2:** Association between systemic immune‐inflammation index and all‐cause mortality in chronic kidney disease.

	Systemic immune‐inflammation index				
	Quartile 1 (<371.19)	Quartile 2 (371.20–522.00)	Quartile 3 (522.01–751.50)	Quartile 4 (>751.50)	*P* value for trend
Unadjusted model	1.09 (0.96, 1.23)	1.00 (Ref)	1.02 (0.89, 1.16)	1.50 (1.34, 1.68)	<.001
Model 1	1.01 (0.89, 1.15)	1.00 (Ref)	1.00 (0.88, 1.13)	1.47 (1.32, 1.63)	<.001
Model 2	0.88 (0.70, 1.10)	1.00 (Ref)	0.85 (0.69, 1.05)	1.23 (1.01, 1.48)	.036

*Note*: Data were presents as hazard ratios and 95% confidence intervals. Model 1 was adjusted for age (continues), sex (male or female), and race/ethnicity (non‐Hispanic White, non‐Hispanic Black, Mexican American, other Hispanic, or others). Model 2 was further adjusted for additional covariates, including BMI (<25, 25–29.9, or 30 kg/m^2^), smoking status (former, current, or never smoker), hypertension (yes or no), diabetes (yes or no), ACR (≥30 or <30 mg/g) or eGFR (continuous), uric acid (≥7 or < 7 mg/dL), cholesterol (≥240 or <240 mg/dL), triglycerides (≥200 or <200 mg/dL), LDL (≥100 or <100 mg/dL), total calcium (continuous), phosphorus (continuous), and total leukocyte count (continuous).

### 3.3 Subgroup analysis

**Figure 3:**
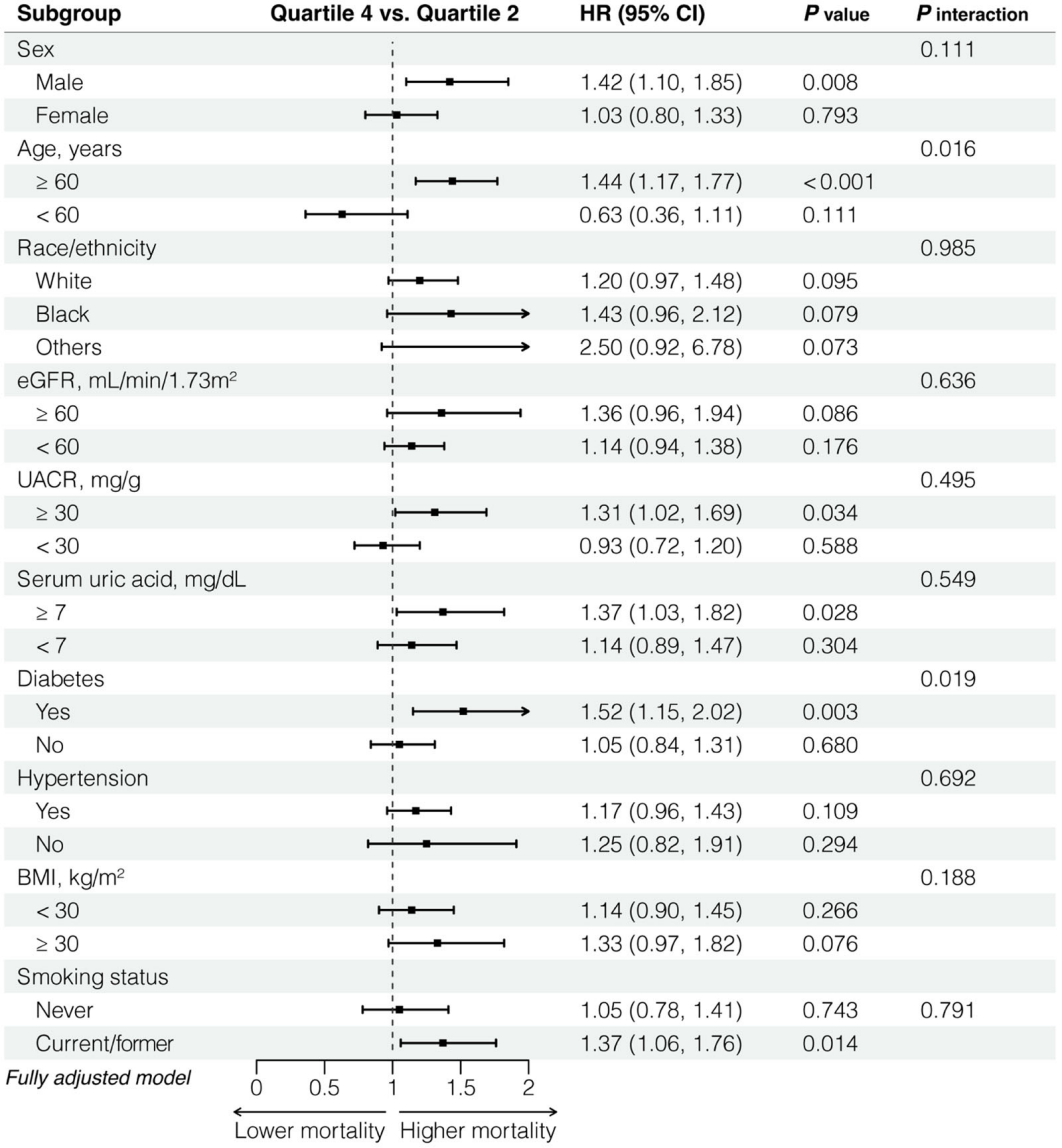
Subgroup analysis of the association between systemic immune‐inflammation index and all‐cause mortality in chronic kidney disease. Comparisons were conducted between Quartile 4 and Quartile 2 using fully adjusted model, adjusting for age (continues), sex (male or female), and race/ethnicity (non‐Hispanic White, non‐Hispanic Black, Mexican American, other Hispanic, or others), BMI (<25, 25–29.9, or 30 kg/m^2^), smoking status (former, current, or never smoker), hypertension (yes or no), diabetes (yes or no), ACR (≥30 or <30 mg/g) or eGFR (continuous), uric acid (≥7 or < 7 mg/dL), cholesterol (≥240 or <240 mg/dL), triglycerides (≥200 or <200 mg/dL), LDL (≥100 or <100 mg/dL), total calcium (continuous), phosphorus (continuous), and total leukocyte count (continuous). BMI, body mass index; CI, confidence interval; eGFR, estimated glomerular filtration rate; HR, hazard ratio; UACR, urinary albumin‐to‐creatinine ratio.

**Figure 4:**
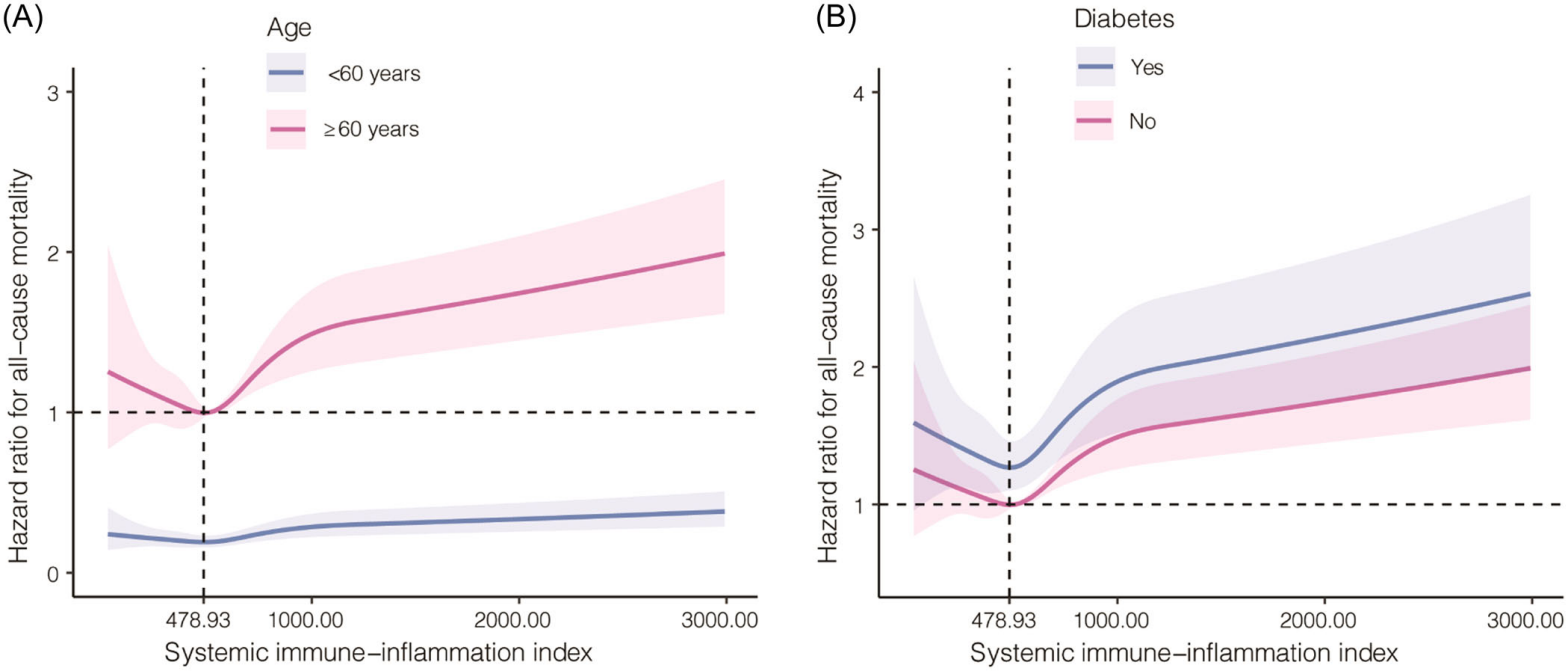
Restricted cubic spline analyses among different subgroups. (A) Subgroups stratified by age; (B) Subgroups stratified by diabetic status.

We conducted subgroup analyses based on sex, age, race, SUA, diabetes, hypertension, BMI and smoking status, as well as different degrees of renal impairment stratified by ACR and eGFR (Figure 3). In participants aged over 60 years, elevated SII values were linked with an increased risk of all‐cause mortality (Model 2: Q4 vs. Q2, HR = 1.44; 95% CI = 1.17–1.77), whereas an opposite result was observed among younger people without statistical significance (Figure 3 and Figure 4A). Moreover, a positive association was unveiled between the higher quartile of SII and all‐cause mortality in individuals with diabetes (Model 2: Q4 vs. Q2, HR = 1.52; 95% CI = 1.15–2.02); however, this relationship failed to achieve statistical significance among those without diabetes (Figure 3 and Figure 4B).

To explore the relationship between renal injury severity and SII, we conducted a detailed analysis based on CKD stages, categorized by eGFR (G1–G5), and albuminuria classifications (ACR: A1, <30 mg/g; A2, 30–300 mg/g; A3, >300 mg/g).^14^ As presented in Table 3, SII exhibited a positive association with albuminuria categories among participants with CKD G3. Patients with CKD G3 A3 classification had a higher proportion in the fourth quartile of SII compared to A1 and A2 categories (proportion of Q4 in A3/A2/A1 categories: 33.77%/26.94%/19.76%). Furthermore, SII demonstrated a positive correlation with CKD stages among participants with ACR categories of A1 and A3. Notably, an elevated SII was associated with a higher risk of all‐cause mortality in participants with CKD categories of G1 A2‐3, G2 A2, G3 A1, G3 A3, and G4 A1‐2 in Model 2.

**Table 3:** Subgroup analysis based on categories of chronic kidney disease stages and albuminuria levels.

eGFR categories (mL/min/1.73 m^2^)		Albuminuria categories (mg/g)			*p* value for different albuminuria levels
		A1 (<30)	A2 (30–300)	A3 (>300)	
G1 (≥90)	No. (death %)	NA	2224 (12.68%)	266 (21.59%)	.008
	SII [Median (IQR)]		528 (369, 751)	552 (380, 796)	.385
	SII (Quartile 1–4 %)		27.07/24.15/25.76/23.02	22.93/25.19/25.19/26.69	.939
	HR per SD (95% CI)		1.23 (1.06, 1.43)	2.57 (1.08, 6.09)	
G2 (60–89)	No. (death %)	NA	1820 (40.15%)	290 (40.34%)	.951
	SII [Median (IQR)]		531 (390, 769)	541 (377, 856)	.519
	SII (Quartile 1–4 %)		26.65/24.56/23.41/25.38	27.58/22.07/23.45/26.90	.814
	HR per SD (95% CI)		1.21 (1.10, 1.34)	1.12 (0.88, 1.41)	
G3 (30–59)	No. (death %)	2966 (40.15%)	965 (56.85%)	305 (60.00%)	<.001
	SII [Median (IQR)]	495 (358, 701)	522 (345, 783)	636 (412, 861)	<.001
	SII (Quartile 1–4 %)	31.12/25.01/24.11/19.76	28.19/21.55/23.32/26.94	19.01/22.30/24.92/33.77	<.001
	HR per SD (95% CI)	1.12 (1.07, 1.17)	1.12 (0.95, 1.33)	1.70 (1.05, 2.75)	
G4 (15–29)	No. (death %)	115 (63.48%)	117 (65.81%)	123 (65.85)	.910
	SII [Median (IQR)]	606 (394, 889)	569 (439, 809)	630 (436, 881)	.257
	SII (Quartile 1–4 %)	16.52/25.22/19.13/39.13	22.22/24.78/26.50/26.50	16.25/23.57/27.46/32.52	.379
	HR per SD (95% CI)	4.18 (1.92, 9.12)	5.24 (1.88, 14.62)	7.60 (0.50, 1.15)	
G5 (<15)	No. (death %)	10 (60.00%)	26 (46.15%)	76 (57.89%)	.562
	SII [Median (IQR)]	1055 (345, 1670)	396 (361, 796)	645 (492, 986)	.354
	SII (Quartile 1–4 %)	30.00/10.00/10.00/50.00	50.00/15.38/11.54/23.08	21.05/13.16/28.95/36.84	.089
	HR per SD (95% CI)	2.45 (0.45, 1.80)	0.78 (0.35, 1.74)	0.97 (0.73, 1.29)	
*p* value for different CKD stages	Mortality	<.001	<.001	<.001	
	SII	.031	.644	.002	
	Quartiles of SII	<.001	.123	.144	

Abbreviations: CI, confidence interval; eGFR, estimated glomerular filtration rate; HR, hazard ratio; IQR, interquartile ranges; NA, not applicable; SD, standard deviation.

### 3.4 Sensitivity analysis

**Figure 5:**
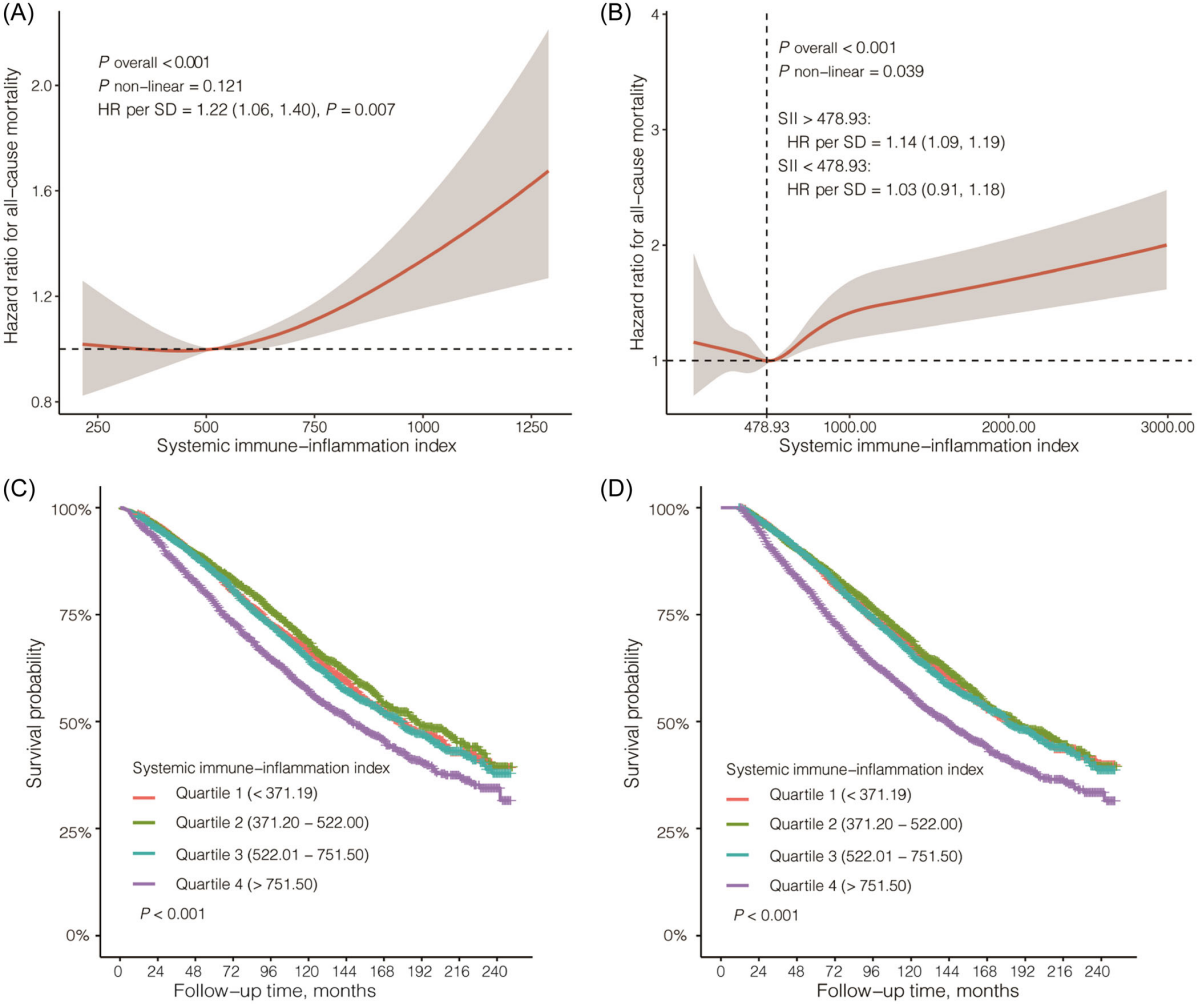
Sensitivity analyses. (A) Sensitivity analysis after removing participants with extreme 5% of systemic immune‐inflammation index; (B) Sensitivity analysis after excluding subjects who died within 1 year after being surveyed; (C) Kaplan Meier curves after after removing participants with extreme 5% of systemic immune‐inflammation index; (D) Kaplan Meier curves after excluding subjects who died within 1 year after being surveyed. HR, hazard ratio; SD, standard deviation.

When the participants with extreme 5% of SII were excluded (<213.63 or >1293.36), SII was found to be positively associated with all‐cause mortality as a linear manner in model 2 among patients with CKD (HR per SD = 1.22; 95% CI = 1.06–1.40, *p* = .007) (Figure 5A,C). After removing participants who died within 1 year of being surveyed, the association between SII and all‐cause mortality showed a similar “J”‐shaped curve, which was consistent with the overall results (Figure 5B,D).

## 4 DISCUSSION

In this cohort study, we observed a “J”‐shaped association between SII level and all‐cause mortality in patients with CKD, with a minimum inflection point of SII level at 478, meaning that the all‐cause mortality initially decreased and then increased with the ascension of SII. Subgroup analyses and interaction tests indicated that the association between SII and all‐cause mortality among participants with CKD was statistically significant in those aged ≥60 years and in diabetic individuals. Inflammation is a prevalent feature observed in CKD, resulting in various detrimental effects, such as glomerulosclerosis, tubular atrophy, vascular impairment, and fibrosis.^31^ In this milieu, standard parameters of inflammatory blood cells, including neutrophils, lymphocytes, platelets, and their derived indices, have been scrutinized for potential associations with untoward CKD outcomes. Notably, the neutrophil‐to‐lymphocyte ratio has been linked to an elevated risk of end‐stage renal disease requiring dialysis, as well as increased all‐cause mortality and cardiovascular mortality among patients with CKD.^32^, ^33^ Similarly, the platelet‐to‐lymphocyte ratio has been associated with an increased risk of all‐cause mortality in patients with CKD.^34^ The SII index, encompassing platelet, neutrophil, and lymphocyte counts, offers a more comprehensive representation of the equilibrium between inflammatory status and host immune response.^35^ In contrast to conventional inflammatory markers, SII more aptly reflected the inflammatory milieu and has demonstrated enhanced prognostic value across several investigations.^9^ Our present findings reveal an association between SII and all‐cause mortality in patients with CKD.

Neutrophils, as the principal immune cells, play a pivotal role in orchestrating the inflammatory response against invading microorganisms and foreign entities. Elevated neutrophil levels were implicated in the pathogenesis and progression of CKD through several mechanisms, including heightened release of reactive oxygen species, protease release contributing to endothelial dysfunction, and increased production of pro‐inflammatory cytokines.^36^ These factors collectively contributed to aggravated kidney injury and the exacerbation of fibrotic changes. A demonstrable increase in neutrophil infiltration has been observed in obstructed kidneys, and neutrophil depletion could decrease the expression of inflammatory factors, inhibit the accumulation of macrophages and suppress renal fibrosis.^37^ Notably, neutrophil gelatinase‐associated lipocalin, produced by neutrophils, has been identified as a marker of interstitial damage and a predictive indicator for the deterioration of kidney function in CKD.^38^ On a similar note, low lymphocyte count has been posited as a marker of malnutrition and inflammation in patients with CKD, with implications for adverse outcomes.^39^ Kim et al. reported an independent association between diminished lymphocyte counts and heightened progression rates in patients with CKD.^40^ Furthermore, Kovesdy et al. documented an inverse relationship between lymphocyte levels and all‐cause mortality among CKD cohorts.^41^ As well as being involved in coagulation, platelets contain a lot of pro‐inflammatory molecules that regulate immune and inflammatory responses. Recent investigations have illuminated the substantial influence of platelets in modulating inflammatory signals and governing leukocyte biology, thereby extending the functionality of the cellular immune system.^42^ Although previous studies have not found significant changes of platelet counts in patients with CKD, impaired function of platelets was indicated.^43^, ^44^ Moreover, clinical evidence has provided insight into platelet activation's contribution to exacerbated kidney impairment and the progression of cardiovascular disorders.^45^, ^46^ Yu et al. have demonstrated an independent association between reduced platelet levels and diminished cardiovascular events among patients with CKD.^47^ Collectively, these evidence indicated that there is a pathological and clinical basis for the association between SII and adverse outcomes in patients with CKD.

The subgroup analyses showed a significant correlation between SII and all‐cause mortality among participants over 60 years old and those with diabetes in the context of CKD. Patients with DKD had higher SII levels versus non‐DKD individuals, as well as the comparison between patients with diabetes and nondiabetic individuals.^11^, ^48^ The relationship between inflammation and diabetes might be bidirectional, which promoting diabetes‐related complications, such as cardiovascular disease and DKD.^49^ Prior investigations have elucidated that platelets exhibit a state of heightened activity, marked by escalated activation, adhesion, and aggregation, stemming from the dysregulation of multiple signaling pathways in individuals with diabetes.^50^ Within this context, vascular inflammation orchestrated by nuclear factor‐κB has been discerned as a key contributor to diabetic milieu.^51^ Aging is typified by systemic, chronic inflammation, which precipitates a spectrum of structural perturbations in kidneys, including fibrosis, as individuals progress in age.^52^ Age‐related modifications within diverse cell types, such as tubular epithelial cells and circulating leukocytes, resulted weakened immune function and an inability to clear inflammatory factors, exacerbating inflammation and ultimately contributing to multiple organ injury among the elderly.^53^ These evidence posited that the convergence of diabetes and aging may engender heightened mortality rates among patients with CKD, due to persistent low‐level inflammation.

The uniqueness of this study lies in the discussion of the staging of CKD, as we attempted to explore the specific differences in the effects of SII within each CKD stage individually. These findings contribute clinical evidence, shedding light on the adverse prognosis of chronic inflammation in the CKD population. The present study underscores the importance of monitoring SII in patients with CKD.

This study boasts several noteworthy strengths. Firstly, it harnessed an expansive data set comprising participants with CKD drawn from a nationally representative population, with meticulous incorporation of appropriate weights and covariates within the statistical models. This rigorous approach augments the credibility of our findings. Additionally, the integration of restricted cubic spline analysis added a layer of depth to the investigation, unraveling the intricate nonlinear associations between SII and all‐cause mortality in participants with CKD. Furthermore, the deployment of comprehensive sensitivity and subgroup analyses lent further credence to the robustness of the results.

The primary limitation of this study stems from its observational design, which means that causality cannot be established. Therefore, caution is needed when interpreting the results. Additionally, the relevant findings currently lack practical application guidance, suggesting that future research should focus on how to apply these findings in practical settings. Furthermore, when applying the results of this study, attention should be paid to potential outliers in SII, as sensitivity analysis showed that extreme values may affect the association between SII and CKD mortality. Additionally, the possibility of unmeasured confounding factors is another limitation, indicating that further research is needed to validate these findings.

## 5 CONCLUSIONS

Using the nationally representative NHANES data, we found a “J”‐shaped association between SII level and all‐cause mortality in patients with CKD. Further research are needed to validate our findings.

## AUTHOR CONTRIBUTIONS

**Meng Jia**: Data curation; formal analysis; validation. **Wenli Yuan**: Data curation; formal analysis; validation; writing—original draft. **Yinqing Chen**: Data curation; formal analysis; validation. **Yi Wang**: Funding acquisition; project administration; supervision; writing—review and editing. **Li Shang**: Conceptualization; writing—review and editing. **Shisheng Han**: Funding acquisition; investigation; methodology; software; visualization; writing—original draft.

## CONFLICT OF INTEREST STATEMENT

The authors declare that they have no competing interests.

## ETHICS STATEMENT

NHANES protocol was approved by the National Center for Health Statistics Ethics Review Board (ethical approval number: NHANES 1999‐2004, #98‐12; NHANES 2005‐2010, #2005‐06; NHANES 2011‐2018, #2011‐17). Details are available at https://www.cdc.gov/nchs/nhanes/irba98.htm.

## Data Availability

The data described in this article can be freely and openly accessed at the NHANES website: https://www.cdc.gov/nchs/nhanes/.
